# A Novel KCNJ2 p.Glu299Ala Variant Associated with Short QT Phenotype and Persistent Atrial Fibrillation in a Child

**DOI:** 10.3390/life16060959

**Published:** 2026-06-05

**Authors:** Gabriela Duica, Eliza Elena Cinteza, Mariana Costin, Tudor Stefan Dulau, Maria Adriana Rasnoveanu, Adelina-Mihaela Sorescu, Alin Marcel Nicolescu, Radu-Gabriel Vatasescu

**Affiliations:** 1 Pediatrics Department, “Carol Davila” University of Medicine and Pharmacy, 041451 Bucharest, Romania; gabriela.antal@drd.umfcd.ro (G.D.);; 2 “Marie S. Curie” Clinical Emergency Hospital for Children, 077120 Bucharest, Romania; 3 Department I-Functional Sciences, Pharmacology, Clinical Pharmacology and Pharmacotherapy, “Carol Davila” University of Medicine and Pharmacy, 050474 Bucharest, Romania; 4 Cardiology Department, “Carol Davila” University of Medicine and Pharmacy, 050474 Bucharest, Romania; radu.vatasescu@umfcd.ro

**Keywords:** short QT syndrome, atrial fibrillation, KCNJ2 mutation, cardiac channelopathy, pediatric patient, genetic arrhythmia

## Abstract

Short QT syndrome (SQTS) is a rare, inherited cardiac channelopathy characterized by an abnormally shortened QT interval, accelerated ventricular repolarization, and an increased risk of atrial and ventricular tachyarrhythmias, including sudden cardiac death (SCD). We report the case of a 14-year-old girl diagnosed with SQTS presenting with persistent atrial fibrillation and a complex independent neurological background. The patient, with no significant family history of cardiac disease or SCD, was incidentally found to have atrial fibrillation and a markedly shortened QT interval during a routine medical evaluation. Although she remained entirely asymptomatic from a cardiovascular perspective, her medical history was notable for maternal Toxoplasma gondii infection during pregnancy, extreme prematurity, and delayed psychomotor development. Electrocardiographic (ECG) findings consistently demonstrated a short QT interval, and genetic testing revealed a likely pathogenic variant in the KCNJ2 gene, consistent with type 3 short QT syndrome (SQTS3). Despite the initiation of antiarrhythmic therapy, atrial fibrillation persisted and the QT interval remained significantly shortened throughout the 24-month follow-up. This case highlights the diagnostic and therapeutic challenges of managing short QT syndrome in pediatric patients, particularly in those who are asymptomatic yet exhibit sustained atrial arrhythmias. It also highlights the coexistence of cardiac channelopathy and neurological comorbidities, emphasizing the importance of a multidisciplinary approach for these distinct clinical entities.

## 1. Introduction

Short QT syndrome is a rare, inherited cardiac channelopathy, transmitted in an autosomal dominant or sporadic manner. It is characterized by an abnormally shortened QT interval, markedly accelerated cardiac repolarization, and an increased susceptibility to atrial and ventricular tachyarrhythmias, predisposing affected individuals to sudden cardiac death [1,2]. Epidemiological studies estimate its prevalence at approximately 0.02–0.1% in the general adult population, and around 0.05% in pediatric cohorts, although the true prevalence remains difficult to establish due to the limited number of genetically confirmed cases worldwide. Corrected QT (QTc) intervals ≤300 ms are observed in less than 3 per 100,000 individuals in large ECG cohorts, yet most such cases do not fulfill the complete clinical criteria for SQTS [3].

The diagnosis of SQTS relies on a combination of clinical presentation, family history, and characteristic findings on the 12-lead electrocardiogram (ECG), although clinical manifestations may be highly variable, ranging from sudden cardiac arrest and syncope to palpitations or a complete absence of symptoms [2]. Among supraventricular arrhythmias, atrial fibrillation (AF) represents a frequent and distinctive feature of SQTS and may be the first or only manifestation, particularly in young patients.

To date, several pathogenic variants involving cardiac ion channel genes have been identified; however, a clear genotype–phenotype correlation has not yet been established, largely due to the limited number of genetically confirmed cases reported in the literature [1,2]. Given the substantial risk of malignant ventricular arrhythmias, placement of an implantable cardioverter-defibrillator (ICD) is considered the cornerstone of therapy in symptomatic patients [4]. Nevertheless, risk stratification and therapeutic decision-making remain challenging in asymptomatic individuals, especially in pediatric patients without a family history of SCD.

Pharmacological treatment options for SQTS are limited, with quinidine currently regarded as the most effective agent for prolonging the QT interval and reducing the arrhythmic burden, although its efficacy may be variable.

In this context, we report the case of a 14-year-old girl with a pre-existing, independent neurological condition, who was entirely asymptomatic from a cardiovascular standpoint and had no relevant family history. Persistent atrial fibrillation associated with a short QT interval was incidentally identified during a routine evaluation and proved refractory to standard medical therapy.

## 2. Case Presentation

A 14-year-old girl was transferred from the Pediatric Nephrology Department to the Cardiology Department following the incidental detection of an arrhythmia during a routine clinical examination. A 12-lead electrocardiogram confirmed atrial fibrillation.

The patient’s medical history was significant for maternal Toxoplasma gondii infection during the first trimester of pregnancy. She was born prematurely at 32 weeks of gestation, with a birth weight of 1400 g and an Apgar score of 5, requiring neonatal intensive care for respiratory distress and poor postnatal adaptation. Subsequent psychomotor development was delayed across multiple milestones.

At 3 years of age, the patient was evaluated for global developmental delay and hypotonic syndrome. Differential diagnoses initially included spinal muscular atrophy and metabolic disorders; however, extensive investigations were negative. Electromyography, electroencephalography, abdominal ultrasonography, echocardiography, ophthalmological examination, and spinal magnetic resonance imaging (MRI) were all within normal limits. Conversely, a brain MRI demonstrated supratentorial demyelinating lesions. Molecular karyotyping revealed no unbalanced chromosomal abnormalities. The patient subsequently underwent a structured physical therapy program, resulting in slow but progressive improvement.

Behavioral disturbances—including auto- and hetero-aggressive behavior, psychomotor agitation, and sleep disorders—prompted treatment with risperidone, levomepromazine, trihexyphenidyl hydrochloride, and carbamazepine. Additionally, the patient required periodic monitoring for neurogenic bladder dysfunction, characterized by alternating urinary retention and incontinence, and experienced recurrent urinary tract infections caused by Escherichia coli and Proteus species.

There was no clinical history, patient reporting, or familial evidence of episodic flaccid muscle weakness or periodic paralysis. The patient’s parents were clinically healthy, with no family history of cardiac or neurological diseases.

## 3. Clinical Findings on Admission

Upon admission to the Cardiology Department, the patient was afebrile and in good general condition. Physical examination revealed a body weight of 53 kg, isolated dysmorphic facial features, hypersalivation, and reduced spontaneous motility. She demonstrated preserved head control but was unable to independently assume a sitting position, though she could maintain it when placed. Significant dorsal kyphosis was also noted. While the patient did not produce meaningful speech, she successfully communicated nonverbally.

Cardiovascular examination revealed irregular heart sounds without audible murmurs, and peripheral pulses were palpable. Vital signs were significant for tachycardia with a heart rate of 150 beats/min; blood pressure was 100/71 mmHg, and oxygen saturation was 96% on room air.

Her background medication regimen included nitrofurantoin, risperidone, carbamazepine, and levomepromazine.

## 4. Investigations

Laboratory investigations—including a complete blood count, serum electrolytes, cardiac enzymes, thyroid function tests, and a coagulation profile—were within normal limits. However, N-terminal pro-B-type natriuretic peptide (NT-proBNP) was elevated at 817 pg/mL (age-specific reference value < 363 pg/mL).

The 12-lead ECG demonstrated atrial fibrillation with a rapid ventricular response (approximately 130 beats/min). Notable repolarization abnormalities included an absent ST segment, with the T wave originating immediately from the S wave, narrow T waves, and a markedly shortened QT interval (QT = 200 ms; QTc = 250 ms via Bazett’s formula). The QT interval was measured manually in leads II and V5 from the onset of the QRS complex to the end of the T wave, utilizing the average of three consecutive beats. Given the elevated ventricular rate, the Fridericia formula was also applied for heart rate correction. The Tpeak–Tend (Tp–Te) interval was 44 ms resulting in a Tp–Te/QT ratio of 0.22. (Figure 1).

Secondary causes of a short QT interval—including electrolyte imbalances (specifically hypercalcemia), acid-base disturbances, hyperthermia, and proarrhythmic drug effects—were excluded.

**Figure 1:**
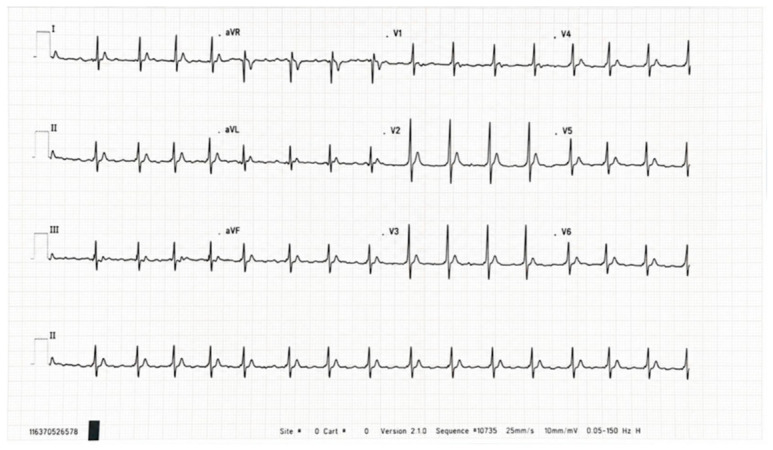
Electrocardiographic findings revealed atrial fibrillation accompanied by a severely abbreviated QT interval.

Transthoracic echocardiography demonstrated that the end of the T wave preceded aortic valve closure by 116 ms; otherwise, findings revealed a structurally normal heart with preserved biventricular systolic function at baseline.

Holter ECG monitoring confirmed persistent atrial fibrillation with a rapid ventricular response (mean heart rate: 136 beats/min) and a consistently shortened QT interval (200 ms). Electrocardiographic recordings obtained from both parents were normal, demonstrating no evidence of QT-interval shortening.

Genetic testing identified a heterozygous pathogenic variant in the KCNJ2 gene (NM_000891.2:c.896A>C, p.Glu299Ala, exon 2), classified as likely pathogenic with a 97% probability of causing short QT syndrome type 3 (SQTS3), based on the mutation’s location in a critical domain and its absence in population databases. Genetic screening targeted the coding regions of six genes associated with short QT syndrome via next-generation sequencing (NGS) on the Illumina platform. Raw sequencing data were aligned against the GRCh38 human reference genome. Single nucleotide variants (SNVs) and copy number variations (CNVs) were identified and processed using the Illumina DRAGEN Bio-IT platform. Downstream variant interpretation was performed using VarSeq vX.X.X analysis software (Golden Helix, Bozeman, MT, USA) and the Human Gene Mutation Database (HGMD) Professional. All identified variants were annotated according to the Human Genome Variation Society (HGVS) nomenclature guidelines using corresponding GenBank accession numbers.

Genetic testing was not performed for the parents due to the absence of clinical or phenotypic manifestations of the disease.

By integrating the patient’s clinical history, electrocardiographic parameters, and genetic findings, application of the Gollob scoring system confirmed a high diagnostic probability of Short QT Syndrome (SQTS) (Table 1).

**Table 1:** The Gollob Scoring System for SQTS diagnosis in our patient.

QTc interval (HR < 100 ms)	<330 ms	3 points
Clinical/family history	negative	0 points
Genotype	positive	1 point

## 5. Treatment and Follow-Up

During hospitalization, oral antiarrhythmic therapy with quinidine was initiated at a dosage of 800 mg/day (200 mg every 6 h) and subsequently up-titrated to 1.6 g/day (400 mg every 6 h). Due to the patient’s body weight, an adult dosing regimen was selected. Despite treatment, no prolongation of the QT interval was observed. Ventricular rate control was achieved by adding metoprolol at 75 mg/day, which reduced the heart rate to 100–110 beats/min. Because the patient remained asymptomatic and hemodynamically stable, neither electrical cardioversion nor catheter ablation was indicated (Table 2).

After 14 days of combined quinidine and metoprolol therapy, transthoracic echocardiography revealed moderate biventricular systolic dysfunction, with a left ventricular ejection fraction (LVEF) of 40% and a right ventricular ejection fraction (RVEF) of 45%. This deterioration was likely secondary to the negative inotropic effects of quinidine, compounded by inadequate heart rate control. Consequently, quinidine was discontinued, and amiodarone therapy was initiated due to its minimal negative inotropic profile compared to other antiarrhythmics and its status as a first-line rhythm control agent for atrial fibrillation. Amiodarone was administered via a 7-day loading protocol, followed by a maintenance dose of 200 mg/day. This therapeutic modification led to the normalization of biventricular systolic function within 7 days and successfully maintained the resting heart rate below 100 beats/min.

Serial 24-h Holter monitoring subsequently demonstrated persistent atrial fibrillation alongside a consistently short QT interval.

Anticoagulation with low-molecular-weight heparin was initially administered for 7 days, followed by acenocoumarol for 10 days, adjusted according to international normalized ratio values. Long-term oral anticoagulation was subsequently discontinued, as the patient lacked classic cardiovascular risk factors. However, given her coexisting neurological substrate and associated behavioral disturbances, an individualized clinical decision was made to initiate antiplatelet therapy as a tailored strategy for stroke risk mitigation and primary thromboembolic prophylaxis.

Catheter ablation was not a viable option due to the high risk of procedure-induced ventricular arrhythmias. Given that the patient achieved complete stabilization, normalized biventricular systolic function, and adequate rate control (<90 beats/min) on a non-invasive regimen of amiodarone and metoprolol, exposing this 14-year-old to a high-risk, low-efficacy invasive procedure was clinically unjustified.

Clinically, the patient presents a low-risk profile characterized by an absence of syncope, ventricular arrhythmias, and familial sudden cardiac death. A comprehensive, multidisciplinary conference involving pediatric cardiologists, electrophysiologists, and the patient’s parents was conducted. Weighing the absence of high-risk ventricular markers against the extreme likelihood of device-related complications given her neuropsychiatric status, a consensus was reached to defer primary prophylactic ICD implantation. Instead, we opted for rigorous, non-invasive long-term rhythm surveillance.

During a two-year follow-up period on stable amiodarone and metoprolol therapy, the patient underwent routine clinical evaluations every 6 months. Serial testing included ECG, echocardiography, 24-h Holter monitoring, and metabolic/endocrine blood panels. The patient remains completely asymptomatic, with stable QT/QTc intervals and permanent atrial fibrillation featuring a controlled ventricular rate (<90 beats/min). No ventricular tachyarrhythmias were documented on serial ambulatory monitoring. Correspondingly, echocardiographic parameters indicate normal ventricular function, consistent with stable, near-normal NT-proBNP levels.

**Table 2:** Chronological Timeline of Clinical Events.

Clinical Phase	Diagnostic Evaluation and Key Findings	Therapeutic Interventions	Clinical Outcomes and Patient Milestones
**Admission**	Incidental detection of irregular rhythm. Hemodynamics: heart rate (HR) 150 beats/min, blood pressure 100/71 mmHg, SpO2 96% on room air.	Hospital admission; continuation of background neuro-psychiatric medications.	Baseline cardiac tracking established; hemodynamic stability preserved.
**Days 2–3 (Diagnostic Workup)**	ECG: Atrial fibrillation, rapid ventricular response (130 bpm), absent ST segment, abruptly short QT/QTc (200/250 ms). TTE: Normal anatomy; T-wave ends 116 ms before aortic valve closure; preserved EF. Labs: NT-proBNP elevated (817 pg/mL).	Secondary acquired causes of short QT excluded. Oral Quinidine initiated at 800 mg/day (200 mg q6h), up-titrated to 1.6 g/day (400 mg q6h). Low-molecular-weight heparin (LMWH) initiated.	No prolongation of QT interval achieved via quinidine monotherapy. Parent ECGs confirmed normal QT intervals.
**Days 4–5**	Persistent atrial fibrillation with continuous rapid ventricular conduction.	Metoprolol (75 mg/day) added to the regimen for rate control.	Ventricular rate brought down to a more stable range (100–110 beats/min).
**Day 14 (Critical Event)**	Follow-up TTE reveals an acute decline in myocardial contractility: LVEF 40% and RVEF 45% (moderate biventricular systolic dysfunction).	Quinidine discontinued due to negative inotropy. Amiodarone introduced via a 7-day loading protocol. Transitioned from LMWH to acenocoumarol.	Drug-induced cardiac dysfunction documented; immediate shift in antiarrhythmic strategy executed.
**Day 21**	Repeat TTE, 24-h Holter, and lab profile. Genetic panel confirms heterozygous c.896A>C (p.Glu299Ala) pathogenic variant in KCNJ2 (SQTS3). Gollob Score: 4 points (High probability).	Amiodarone adjusted to 200 mg/day maintenance dose. Anticoagulation discontinued. Antiplatelet therapy initiated for neurological risk mitigation.	Biventricular systolic function fully normalized within 7 days. Resting heart rate controlled below 100 beats/min. Discharged to outpatient status.
**Months 6, 12, 18, & 24 (Longitudinal Surveillance)**	Semi-annual check-ups: Serial ECGs, 24-h ambulatory Holter recordings, TTE, and organ function panels (liver, renal, thyroid, electrolytes, NT-proBNP).	Maintained continuous, stable, long-term therapeutic regimen of Amiodarone and Metoprolol.	Complete clinical tolerance. Persistent AF with well-controlled ventricular rate (<90 bpm). Preserved biventricular function; near-normal NT-proBNP titers. Zero ventricular arrhythmias. Prophylactic ICD deferred.

## 6. Discussion

Short QT syndrome is an exceptionally rare inherited cardiac channelopathy, with fewer than 100 clinically and genetically well-characterized cases reported to date. All affected individuals described in the literature present with a markedly shortened QT interval, typically below 320 ms, in the absence of structural heart disease [5]. Atrial fibrillation frequently represents the initial clinical manifestation of SQTS and may occur in pediatric cohorts, often preceding the development of ventricular arrhythmias.

The *KCNJ2* gene encodes the inward rectifier potassium channel Kir2.1, which plays a crucial role in maintaining the resting membrane potential and the terminal phase of cardiac repolarization [6,7,8]. The inward rectification property of Kir2.1 is mediated by voltage-dependent blockade via intracellular Mg^2+^ ions and polyamines, relying on negatively charged residues located both in the transmembrane domain (D172) and the cytoplasmic C-terminal region (E224, D255, D259, and E299) [9,10,11,12,13,14]. Pathogenic variants affecting these specific sites disrupt channel rectification, profoundly altering cardiac electrophysiology.

To date, four missense mutations in *KCNJ2*—D172N, E299V, M301K, and K346T—have been associated with the SQTS type 3 (SQTS3) phenotype [6] (Table 3). In the present report, we describe a novel heterozygous mutation, p.Glu299Ala (E299A), identified in a 14-year-old girl presenting with SQTS and permanent AF. This mutation affects the same highly conserved amino acid residue as the previously described E299V variant, replacing glutamic acid with alanine, a neutral amino acid, thereby altering the electrostatic properties of the Kir2.1 channel [15,16,17,18,19,20,21].

A comparable case was reported by Deo et al. in 2013 involving an 11-year-old boy harboring the heterozygous *KCNJ2* E299V mutation, who presented with paroxysmal AF and a markedly shortened QT interval [6]. The phenotypic similarities between that patient and ours—including early-onset AF, the absence of structural heart disease, and a negative family history—support the pathogenic relevance of substitutions at position 299 of the Kir2.1 channel.

Experimental and computational studies have demonstrated that replacing glutamic acid at position 299 with a neutral residue not only reduces inward rectification of the inward rectifier potassium current IK1 but also generates an abnormal outward current during depolarization, a phase in which the channel is normally inactive. This results in a marked abbreviation of the action potential duration (APD) and manifests clinically as an extremely short QT interval [6]. Furthermore, E299 substitutions impair rate-dependent adaptation of repolarization, a phenomenon also observed in our patient, whose QT interval remained consistently short regardless of the heart rate.

Simulations conducted by Deo et al. and later by Whittaker et al. demonstrated that both homozygous and heterozygous forms of the p.Glu299Val (E299V) mutation significantly shorten the atrial APD and create a substrate for re-entry, explaining the high susceptibility to AF [6,22]. Similar mechanisms are likely involved in the arrhythmogenic phenotype observed in our patient, who carries the p.Glu299Ala (E299A) variant.

The clinical and electrophysiological observations associated with known *KCNJ2* mutations in SQTS3 patients are summarized in Table 3 [23].

Therapeutic options for patients with SQTS3 remain strictly limited. Amiodarone has been shown to control AF in E299V mutation carriers primarily through rapid delayed rectifier potassium current IKr blockade, while computational models suggest that partial Ikr inhibition may destabilize re-entrant circuits, particularly within the pulmonary vein region [6,22]. Other agents, such as chloroquine, which blocks both IK1 and IKr currents, have demonstrated efficacy in experimental models, though clinical translation data remain scarce [14,23,24,25,26]. Despite these theoretical considerations, pharmacological therapy in our patient failed to restore sinus rhythm or prolong the QT interval, highlighting the limited and unpredictable response to antiarrhythmic drugs in SQTS.

Given the malignant arrhythmic potential of SQTS, implantable cardioverter-defibrillator therapy represents the cornerstone of management in high-risk patients. However, ICD implantation in pediatric and asymptomatic individuals poses significant challenges, including technical difficulties, psychological burden, and a high incidence of inappropriate shocks due to T-wave oversensing and concomitant AF. Current guidelines strongly recommend ICD implantation in survivors of sudden cardiac arrest or in patients with documented sustained ventricular arrhythmias, but risk stratification in asymptomatic patients remains challenging [1,3,27,28,29].

The optimal long-term management strategy for persistent AF in a 14-year-old patient with congenital SQTS3 demands a careful balance between rhythm control efficacy and procedural safety. The risk of procedure-induced ventricular arrhythmias represents a significant clinical barrier to invasive intervention in SQTS3. Given the pro-arrhythmic risks and the low probability of maintaining long-term sinus rhythm, a conservative, pharmacologically driven rate-control strategy is highly favored.

In the absence of robust risk stratification tools and long-term outcome data, the management of asymptomatic pediatric patients with SQTS should be highly individualized, balancing the potential benefits of ICD implantation against device-related complications. Our case further underscores the unmet need for genotype-specific therapeutic strategies and long-term multicenter registries to better define optimal management in this rare but potentially lethal condition.

## 7. Conclusions

This case underscores the diagnostic and therapeutic challenges posed by short QT syndrome in pediatric patients, particularly when the disease manifests as permanent atrial fibrillation in the absence of prodromal cardiovascular symptoms or a positive family history. The identification of a novel KCNJ2 mutation, p.Glu299Ala (E299A), associated with short QT syndrome type 3, expands the spectrum of pathogenic variants affecting the K2.1 channel and further highlights the marked phenotypic variability of this condition.

Despite targeted antiarrhythmic therapy, rhythm control could not be achieved, and the QT interval remained markedly shortened, underscoring the limited and unpredictable efficacy of currently available pharmacological strategies in SQTS. In asymptomatic pediatric cohorts, the absence of validated risk stratification tools complicates decisions regarding implantable cardioverter-defibrillator therapy, necessitating a highly individualized, patient-centric approach.

In conclusion, this case highlights the paramount importance of comprehensive genetic evaluation, rigorous long-term follow-up, and multidisciplinary management in pediatric SQTS. Further clinical and translational research remains essential to refine risk assessment models, establish genotype-specific therapeutic options, and clarify long-term outcomes in this rare but potentially life-threatening channelopathy.

## Figures and Tables

**Table 3:** Summary of clinical and genetic characteristics of reported KCNJ2 (SQTS3) variants.

Genotype	Protein Domain & Location	Key Electrocardiographic Parameters	Clinical Phenotype & Neurodevelopmental Features	Inheritance Pattern & Family Screening
**D172N**	Transmembrane helix M2	QTc 315–320 ms; narrow, peaked T waves.	Asymptomatic in child; presyncope and palpitations since age 15 in father.	*De novo* mutation confirmed via genetic analysis.
**E299V**	C-terminus cytoplasmic region	Extremely short QTc (200 ms); absent ST segment; QRS-T wave merger.	Paroxysmal atrial fibrillation episodes documented on Holter monitoring.	Parents negative for the mutation.
**M301K**	C-terminus cytoplasmic region	Extremely short QTc (194 ms).	Paroxysmal atrial fibrillation; inducible ventricular fibrillation; severe intellectual disability and epilepsy.	Family members evaluated with normal QTc intervals.
**K346T**	C-terminus cytoplasmic region	QTc 331 ms; narrow, peaked T waves.	Identified in 9-year-old identical twins presenting with epilepsy and autism spectrum disorder.	Heterozygous variant also identified in the asymptomatic mother.

## Data Availability

The original contributions presented in this study are included in the article. Further inquiries can be directed to the corresponding author.
